# Frequency of Depression-Related Symptoms in Caregivers of Patients with Silicosis

**DOI:** 10.1155/2019/6035920

**Published:** 2019-02-11

**Authors:** Jianjun He, Weirong Dai, Ying Li, Li He, Ruixue Huang

**Affiliations:** ^1^Changsha Medical University, Changsha, China; ^2^Hunan Province Hospital of Occupational Disease Prevention and Control, 410073 Changsha, China; ^3^Department of Occupational and Environmental Health, Xiangya School of Public Health, Central South University, 410078 Changsha, China

## Abstract

**Background:**

Few studies have evaluated depression in female caregivers of patients with silicosis. Thus, the aim of this study was to estimate the prevalence of depression in such caregivers and to clarify the factors associated with symptoms of depression.

**Methods:**

Depressive symptoms were assessed using the Center for Epidemiologic Studies Depression Scale (CES-D).

**Results:**

A total of 561 participants met the inclusion criteria and were enrolled in the study. The mean CES-D score was 16.68, with a standard deviation (SD) of 8.57; the sex-classified analysis indicated that the mean CES-D score of female caregivers was 17.79 (SD: 10.17), while the mean score of male caregivers was 14.98 (SD: 8.36) (*p* < 0.05). 68.6% caregivers who were beyond the cutoff score (16) with the following factors were more likely to report depression-related symptoms: unemployed status (OR = 1.752, 95% CI: 1.35–2.01, *p*=0.032) and caregiver for more than 48 months (OR = 1.26, 95% CI: 1.61–2.43, *p*=0.027).

**Conclusions:**

Collectively, there is statistical difference between female caregivers of patients with silicosis and male ones. More effort is needed to meet the psychosocial needs of these caregivers.

## 1. Introduction

Silicosis is a well-known disease caused by the inhalation of free crystalline silicon dioxide or silica, with the main symptom being pulmonary fibrosis [1]. Exposure to free crystalline silicon dioxide or silica continues to affect workers' health globally. According to Occupational Safety and Health Association (OSHA) compliance data (1979–1995), more than 121,000 workers in the United States have been exposed to at least 10 times the recommended exposure limit (REL) of 0.05 mg/m^3^ of these substances [2]. Between 1987 and 1996, there were approximately 3,600 to 7,300 silicosis cases per year and 2,787 deaths attributed to silicosis-related causes [3]. More than 2.3 million workers are estimated to be currently exposed to silica, not only due to the emergence of new occupations such as extracting natural gas but also some ancient occupations such as mining [4]. During the early 1990s, 3.2 million workers were estimated to have been exposed to crystalline silica in the European Union [5]. In Sweden, 111 deaths were attributed to silicosis between 1997 and 2013 [6].

Workers in low- and middle-income countries are the major victims of silicosis. In 1999, the Indian Council of Medical Research reported that silicosis is a common occupational disease across India [7]. Viswanathan et al. assessed workers in an Indian Ordnance Factory and found that the prevalence of silicosis was 3.4% [8]. China has one of the largest silicosis patient populations worldwide. Over the past three decades, approximately 660,000 cases of pneumoconiosis have been confirmed, of which 90% were silicosis, and the number has gradually decreased every year [9]. Han et al. found that almost all silicosis patients experience symptoms of anxiety and depression [10]. Silicosis patients in Turkey have also been found to have increased anxiety and depressive symptoms [11]. These symptoms may be related to common comorbidities such as tuberculosis, pulmonary infection, spontaneous pneumothorax, and pulmonary heart disease [12]. Additionally, patients with silicosis may experience a loss of independence. Previous studies have focused primarily on the mental health status of patients with silicosis, most of whom are male; however, the mental health status of family members, particularly female family members who become caregivers, has seldom been investigated. In a recent meta-analysis, Sallim et al. found that female caregivers of patients with Alzheimer's disease were 1.53 times more likely to have depression than male caregivers (95% CI: 1.29–1.83) [13]. Additionally, female caregivers have been found to have higher depression levels when the patients are disabled males [14]. We hypothesize that female family members are more prone to depressive symptoms than their male counterparts. Depression may lead to physical pain and suicidal behaviors; thus, it is important to understand factors related to depression in female caregivers of patients with silicosis. In this study, a survey on depressive symptoms was used to evaluate female family members who provide care for patients with silicosis. The social and economic factors that may predict high risk for depressive symptoms in female caregivers were explored. The study aimed to provide the basic information necessary to identify interventions for mitigating depression in female caregivers.

## 2. Methods and Materials

### 2.1. Study Design and Participants

Hunan Province is known for its rich mineral resources and has a long history of mining. It has been named as the “home of nonferrous metals.” Workers in the mining industry are often exposed to large amounts of silica. According to the health news website, there are 41,386 patients with silicosis in Hunan Province, the highest number among all Chinese provinces (http://kns.cnki.net/KCMS/detail/detail.aspx?FileName=JIKA200705080072&DbName=CCND2007). A cross-sectional survey was conducted in the affiliated hospital of Occupational and Disease Control Institute in Hunan Province, China, which provides occupational examination and diagnostic services. We included family caregivers of patients who met the diagnostic criteria for silicosis; the included caregivers had been providing care for at least 1 month. Participants were recruited from July 1, 2016, to Sep 1, 2017, and all agreed to take part in the survey.

### 2.2. Instrument

The Center for Epidemiologic Studies Depression Scale (CES-D) was used to assess the depressive status of the caregivers. The CES-D measures the frequency of common depressive symptoms in the previous week. The scale contains 20 items, each of which is scored from 0 to 3 (0 = never, 1 = sometimes, 2 = frequently, and 3 = always), with the overall score ranging from 0 to 60. A score of 16 or higher is generally taken to indicate clinically meaningful depression [15]. The CES-D is a validated and widely used instrument suitable for measuring depressive symptoms in the general population. The CES-D has also been used to measure depressive symptoms of mothers who care for children with mental health problems [16]. The Chinese version of the CES-D has good reliability and validity and is widely used in epidemiological surveys [17]. In the current study, data on the participants' age, occupation, ethnicity, occupational history, and other socioeconomic factors were also collected. The female caregivers were divided into two groups (a depressive group and a normal group) categorized according to the CES-D (Chinese version) cutoff value for depression of 16. The study was approved by the Xiangya School of Public Health IRB (XYGW-2017-45). The survey was conducted using face-to-face interviews. All enrolled participants were provided with information about the survey (delivered orally), and all were informed that they could withdraw at any time.

### 2.3. Statistical Methods

Continuous data are presented as means ± standard deviation (SD). Descriptive statistics are presented as means ± SD and as frequencies. Descriptive statistics were generated, including general participant characteristics and the level of depression. Analysis of *Χ*^2^ test was used to compare difference in categorical data between the depressive and normal groups. Analysis of variance and *t*-tests were used to compare differences in depression scores between the depressive and normal groups. Multiple linear regression was used to analyze factors influencing the level of depression in the caregivers. Statistical analyses were conducted using SPSS software (ver. 23.0; SPSS Inc., Chicago, IL, USA), and statistical significance was set at *p* < 0.05 (two-tailed test).

## 3. Results

In total, 570 family members satisfied the inclusion criteria, of whom 3 declined to complete the survey. Six participants were excluded due to missing data (>20%). Thus, the final sample consisted of 561 (96.7%) participants. The age range was 30 to 79 years (mean age: 52.6 ± 19.7 years). The sociodemographic characteristics of the participants are presented in Table 1. The mean CES-D score was 16.68 (SD: 8.57); 385 participants (68.6%) scored above the cutoff of 16 and thus were included in the depressive symptoms group. The remaining participants (*n*=176; 31.4%) were included in the normal group. CES-D scores differed significantly between the two groups. As shown in Table 1, unemployed status, low yearly income, long duration of caregiving, and low education level had a significant association with depressive symptoms (compared to employed status, high yearly income, short duration of caregiving, and high education level, respectively). The sex-classified analysis indicated that the mean CES-D score of female caregivers was 17.79 (SD: 10.17) while male caregivers was 14.98 (SD: 8.36). After conducting *t*-test, there is a significant difference between two groups (*p* < 0.05). The CES-D measures the frequency of common depressive symptoms. Female caregivers with a lower yearly income were more likely to report depressive symptoms compared to those with a higher income (OR = 1.45, 95% CI: 1.17–2.08, *p*=0.005; OR = 1.92, 95% CI: 1.63–3.41) (Table 2), as were those who were unemployed versus employed (OR = 1.752, 95% CI: 1.35–2.01, *p*=0.032), those who had been caregivers for more than 24 months versus less than 24 months (OR = 1.12, 95% CI: 1.05–1.57, *p*=0.039; OR = 1.26, 95% CI 1.61–2.43, *p*=0.027), and those with a low versus high education level (OR = 2.09, 95% CI: 1.77–3.53, *p*=0.04).

## 4. Discussion

In our sample of 361 female caregivers, the mean CES-D score was 17.68 ± 8.57 and 175 (67%) caregivers were above the cutoff score for depression of 16. The results show that female caregivers of silicosis patients present with severe depressive symptoms; thus, there is a need for interventions to improve their mental health status.

We focused our attention on female caregivers for several reasons. First, in China, with rapid industrialization, an increasingly large number of small mines, factories and heavy metal industries, producing various types of dust, have appeared. These industries recruit internal migrant workers since they have no specific educational requirements. As a result, the number of silicosis cases has dramatically increased in recent years. According to one report, almost 90% of silicosis patients are male [18]. Specifically, the internal migrant workers are almost always male [9]. Thus, their family members, including wives, mothers, and other female relatives, are the most likely caregivers. Second, some previous reports have indicated that the incidence of depression is higher among females specifically versus the general population. Derajew et al. reported a 25% higher rate of depression among female primary caregivers versus the general population [19]. Tong et al. investigated the mental health status of 13,425 female nurses and found that mental health problems, in particular, depressive symptoms, tended to be severe [20]. Rahmani et al. reported that female spousal caregivers of husbands with severe mental illness were themselves prone to mental health problems [21]. Menon et al. demonstrated that 74.62% of the caregivers of stroke survivors were female and also that female caregivers had higher levels of psychological stress and sleep disturbance than male caregivers [22]. Third, female caregivers of patients with silicosis represent a highly specific group, but one that is estimated to include several million people. However, no previous studies have specifically focused on the mental health status of this population. Thus, in this study, we conducted a survey to address this gap.

Our study showed that more than 63% female caregivers had an education level of junior high school or lower, 61% were unemployed, 36% had been caregivers for more than 24 months, and 63.9% had an annual income under 10,000 RMB yuan. According to previous reports [9], patients with silicosis are typically internal migrant workers from rural or remote areas and their wives are almost always fellow villagers who thus also reside in rural or remote areas. Accordingly, their education levels and annual incomes are very low. A study by Yu et al. indicated that the education level was primary school or less for more than 39% of the Chinese rural population, while more than 45% had an annual income per person under 150 RMB yuan [23]. Our previous study showed that, even among village doctors, the education level was low: more than 26% had an education level under junior school, and more than 30% had an annual income of 10,000 RMA yuan or less [24]. In this study, the income level of the female caregivers was lower than in previous studies. This may be because patients with silicosis often experience a severe economic burden. It was reported that, in rural China, the number of people who were unemployed was significantly higher than the number who were employed (*t*=−1.60) [23]. Unemployed female caregivers of patients with silicosis are under severe economic stress, needing to meet the costs of daily care and medicines. Silicosis has been identified as an irreversible disease in which the development of the lung fibrosis still continues even after disengagement of dust exposure. Importantly, with disease progression, the severe and common complication of tuberculosis can eventually lead to death [25]. Thus, female caregivers often have to not only buy medicine for prevention of silicosis but also for treating tuberculosis, representing an expense that exceeds their income. A large number of studies have identified a low income as a risk factor for mental health problems. Lee et al. reported that low income was a significant risk factor for depression in patients with chronic obstructive pulmonary disease (COPD) (OR = 2.17, 95% CI = 1.55–3.04, *p* < 0.01) [26]. Katzan et al. demonstrated that a lower income and female sex were associated with worse median scores on the modified Rankin Scale (mRS) following ischemic stroke [27]. Duration of caregiving was another risk factor for depression in our study, consistent with previous studies. Khan et al. reported that depression in HIV caregivers was positively associated with duration of caregiving [28]. These data indicate that higher depression levels among females are associated with a low income, low education level, and long duration of caregiving. The gaps between mental health and these predictors among female caregivers of patients with silicosis require strong motivation to enhance the female population via public health campaigns.

This study had some limitations. First, the design was cross-sectional, and therefore, health outcomes and causality could not be determined. Second, there was only one outcome measure; other measures, including life satisfaction and self-efficacy, could further our understanding of female caregivers' mental health. Third, our study did not compare the female caregivers with their male counterparts, nor with the general population. Future investigations may benefit from expanding the study design used herein, or from applying additional measures to better capture mental health status in this population.

Collectively, there is a statistical difference between female caregivers of patients with silicosis and male ones. Since the mean score in the female group was over 16, more efforts are needed to improve public awareness of depression in this group, and large-scale public health interventions are required. Low income, unemployed status, long duration of caregiving, and low education level are risk factors for depression; thus, providing more employment and education opportunities could be key for mitigating the disorder.

## Figures and Tables

**Table 1:** Sociodemographic characteristics of the participants (*n*=561).

Variables	*n*	Percent	Normal group (scores < 16) (*n*=176) (%)	Depression group (scores ≥ 16) (*n*=385) (%)	*p* value
Sex					**0.04** ^*∗*^
Male	200	35.7	101 (57.4)	99 (25.7)	
Female	361	64.3	97 (43.6)	264 (74.3)	

Family annual income					**0.03** ^*∗*^
RMB < 10000	197	35.1	60 (34.1)	137 (35.6)	
10001–30000	262	46.7	80 (45.5)	182 (47.2)	
>30001	102	18.2	36 (20.4)	66 (17.2)	

Alcohol habit					0.47
Never	190	33.8	98 (55.7)	92 (23.9)	
<2 times per month	242	43.1	13 (7.3)	229 (59.5)	
1-2 times per week	66	11.7	33 (18.7)	33 (8.57)	
>3 times per week	63	11.4	32 (18.3)	31 (8.03)	

Exercise habit					0.38
No exercise	145	25.8	70 (39.8)	75 (19.5)	
1-2 times per week	212	37.8	50 (28.4)	162 (42.1)	
3-4 times per week	118	21.0	30 (17.0)	88 (22.8)	
>5 times per week	86	15.4	26 (14.8)	60 (15.6)	

Care duration (months)					**0.04** ^*∗*^
1–12	246	43.9	70 (39.8)	176 (45.7)	
12–48	190	33.9	86 (48.9)	104 (27)	
>48	125	22.2	20 (11.3)	105 (27.3)	

Educational level					**0.02** ^*∗*^
Under junior high school	366	65.2	101 (57.4)	265 (68.9)	
Beyond junior high school	195	34.8	75 (43.6)	120 (31.1)	

Religion^*#*^					0.17
No	418	74.5	106 (61.2)	312 (81)	
Yes	143	25.5	70 (39.8)	73 (19)	

Occupation					0.02^*∗*^
Employed	390	69.5	116 (65.9)	274 (71.2)	
Unemployment	171	30.5	60 (34.1)	111 (28.8)	

^*#*^A cultural system of designated behaviors, ethics, or organizations that relates humanity to supernatural or spiritual elements such as Buddhist. ^*∗*^*p* < 0.05.

**Table 2:** Predictors of depressive symptoms for the female caregivers.

Variables	OR	95% confidence limits	*p*
Annual income (RMB yuan)			
>30001	1		
10001–30000	1.45	(1.17, 2.08)	0.005^*∗*^
<10000	1.92	(1.63, 3.41)	0.008^*∗*^

Occupation			
Employed	1		
Unemployed	1.752	(1.35, 2.01)	0.032^*∗*^

Care duration (months)			
1–12	1		
12–48	1.12	(1.05, 1.57)	0.039^*∗*^
>48	1.26	(1.61, 2.43)	0.027^*∗*^

Educational level			
Beyond junior high school	1		
Under junior high school	2.09	(1.77, 3.53)	0.039^*∗*^

OR: odds ratio, CI: confidence interval, ^*∗*^demonstrates significance at the 0.05 level.

## Data Availability

The data that support the findings of this study are available from Central South University, Hunan Province Occupational Disease Control and Prevention, but restrictions apply to the availability of these data, which were used under license for the current study and so are not publicly available. Data are however available from the authors upon reasonable request and with permission of Central South University, Hunan Province Occupational Disease Control and Prevention.
